# A case of a thoracic mass negative on thoracentesis diagnosed by cryobiopsy from the visceral pleura

**DOI:** 10.1002/rcr2.1050

**Published:** 2022-10-17

**Authors:** Chie Morita, Atsushi Kitamura, Katsuhito Kinoshita, Kuniyo Sueyoshi, Manabu Murakami, Shosei Ro, Ryosuke Imai, Kohei Okafuji, Fumitsugu Kojima, Yutaka Tomishima, Torahiko Jinta, Toru Bando, Naoki Nishimura

**Affiliations:** ^1^ Department of Respiratory Medicine, Thoracic Center St. Luke's International Hospital Tokyo Japan; ^2^ Department of Respiratory Medicine National Center for Global Health and Medicine Tokyo Japan; ^3^ Department of Thoracic Surgery, Thoracic Center St. Luke's International Hospital Tokyo Japan; ^4^ Department of Anesthesia and Intensive Care Unit St. Luke's International Hospital Tokyo Japan

**Keywords:** cryobiopsy, thoracoscopy, visceral pleura

## Abstract

Thoracoscopy under local anaesthesia is recommended for malignant tumours with negative pleural effusion cytology. Cryobiopsy from the visceral pleura by thoracoscopy under local anaesthesia can provide more diagnostic options for patients with thoracentesis‐negative malignant effusions. Here we present the first case in which this technique was used. The patient had a pleural metastasis that could not be diagnosed even with rapid cytology of the parietal pleura biopsy. Indications, technical pitfalls, and safety tips are discussed.

## Graphical abstract

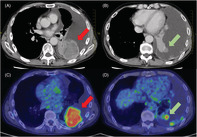

## INTRODUCTION

According to the British Thoracic Society (BTS) guidelines, malignant pleural effusions are diagnosed by thoracentesis in approximately 60% of cases, and thoracoscopy under local anaesthesia is considered to have a diagnostic yield as high as that of video‐assisted thoracoscopic surgery in pleural effusions that are negative on thoracentesis.^1^ In thoracoscopy under local anaesthesia, biopsies are usually taken from the parietal pleura. However, Terashita et al. safely performed a forceps biopsy from the visceral pleura during thoracoscopy under local anaesthesia and diagnosed lung adenocarcinoma.^2^

Here we report a case of a patient who was negative on thoracentesis and underwent cryobiopsy from the visceral pleura by thoracoscopy under local anaesthesia. The technique was performed safely and led to a diagnosis.

### Case report

**FIGURE 1:**
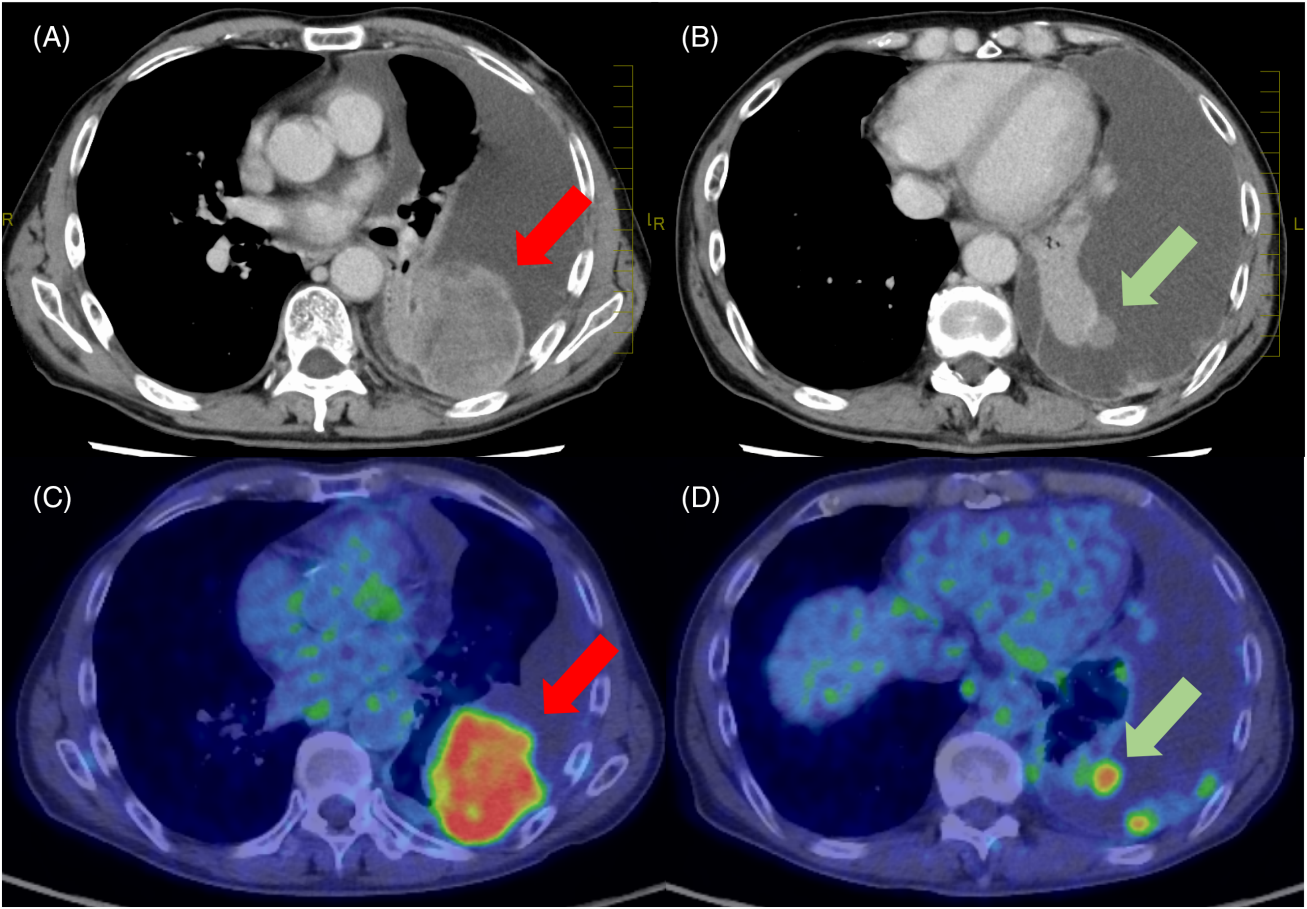
(A) Computed tomography (CT) image showing pleural effusion and a 65‐mm large mass (red arrow). (B) A 20‐mm convex nodule extending into the thoracic cavity from the visceral pleura (green arrow). (C, D) Positron emission tomography‐CT images showing fluorodeoxyglucose accumulation in each mass (red and green arrows)

**FIGURE 2:**
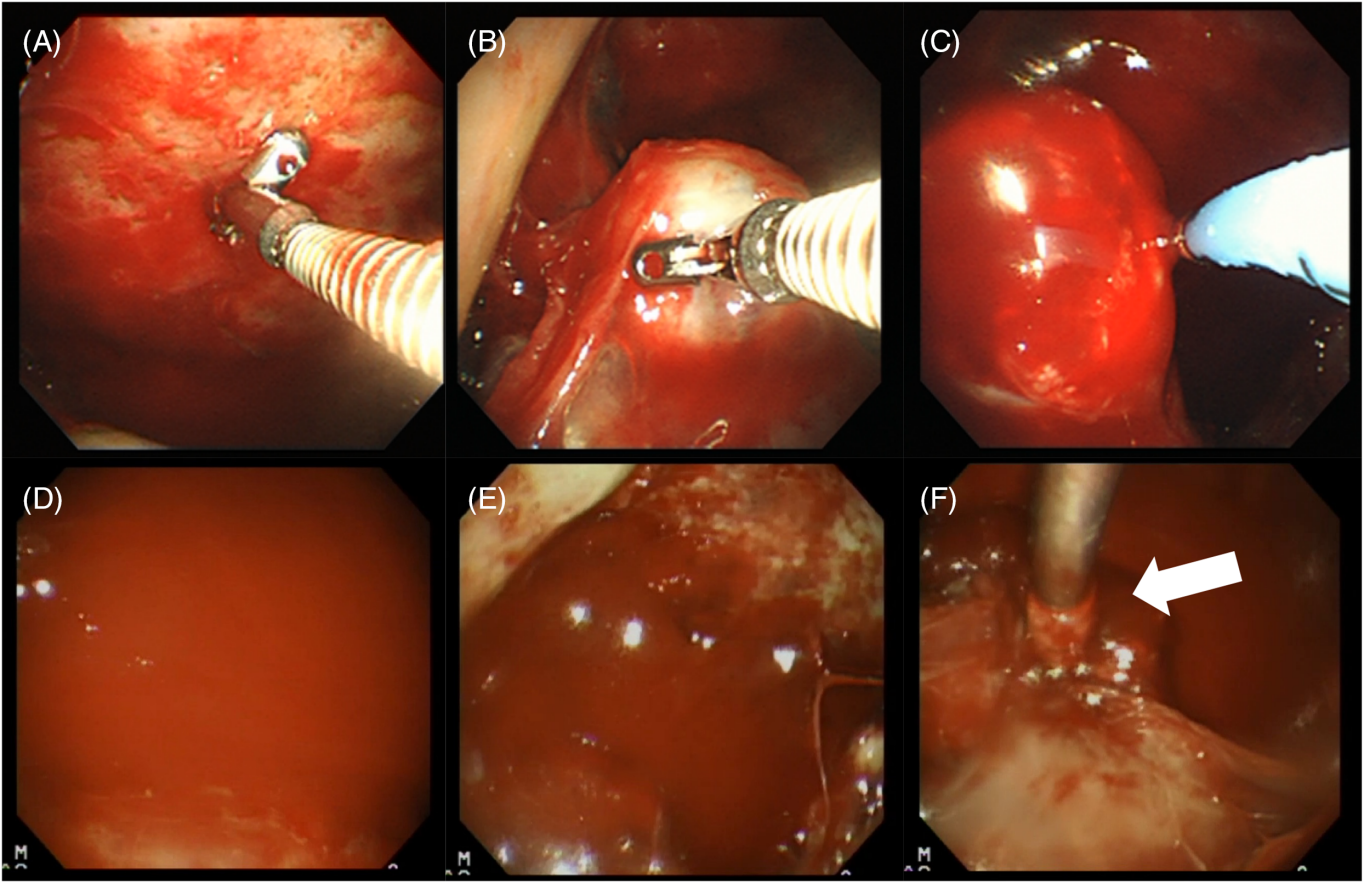
(A) The parietal pleura was thickened. A forceps biopsy was obtained from the pleural wall and was negative on rapid cytology. (B) For the convex mass extending into the thoracic cavity from the visceral pleura, we grasped the forceps vertically to obtain a biopsy specimen. (C) For the mass protruding into the thoracic cavity, we pressed the cryoprobe tangentially on the side of the mass and froze it for 3 s to obtain a biopsy. (D) After cryobiopsy, the biopsy site bled and was compressed by thoracoscopy. (E, F) A small amount of bleeding persisted despite thoracoscope compression, so the cardiothoracic surgeon created a second port and inserted a cotton swab (Naruke Thoraco Cotton, Kenzmedico, Saitama, Japan; white arrow) to stop the bleeding

A 65‐year‐old man with Parkinson's disease, post‐total resection for left renal carcinoma, cryotherapy for right renal carcinoma and postoperative right breast carcinoma, presented to St. Luke's International Hospital complaining of dyspnoea. Chest computed tomography (CT) and positron emission tomography–CT showed an intrathoracic mass, nodules on the parietal and visceral pleura, mild pleural thickening and pleural effusion (Figure 1). The maximal SUV‐max was 9.8. Left thoracentesis was negative for cytology. The patient was an elderly and had Parkinson's disease, and we decided to perform thoracoscopy under local anaesthesia, which is a less invasive procedure. A cardiothoracic surgeon was also present for the examination because we expected a high amount of bleeding if the mass was a recurrence of renal cancer. We created a port in the left side of the chest and inserted a thoracoscope (LFT‐260; Olympus, Tokyo, Japan). Due to encapsulated pleural effusion, we could not obtain a clear view (Figure 2A). We first obtained 10 forceps biopsies from the parietal pleura, but rapid cytological evaluation was negative. Therefore, we obtained five forceps biopsies from the visceral pleural mass protruding into the thoracic cavity (Figure 2B). Next, a flexible cryoprobe, 1.9 mm in diameter (ERBOCRYOCA; ERBE, Tubingen, Germany), was applied tangentially to the mass and the mass was frozen for 3 s for one biopsy (Figure 2C). After the cryobiopsy, there was a small amount of bleeding (Figure 2D), so compression was performed twice with a thoracoscope (Figure 2E). Because there was a small amount of persistent bleeding, a second port was created by a cardiothoracic surgeon and a cotton swab (Naruke Thoraco Cotton; Kenzmedico, Saitama, Japan) was inserted from the second port to stop the bleeding under thoracoscope observation (Figure 2F). The drain was removed the next day, and the patient was discharged 2 days after the examination.

**FIGURE 3:**
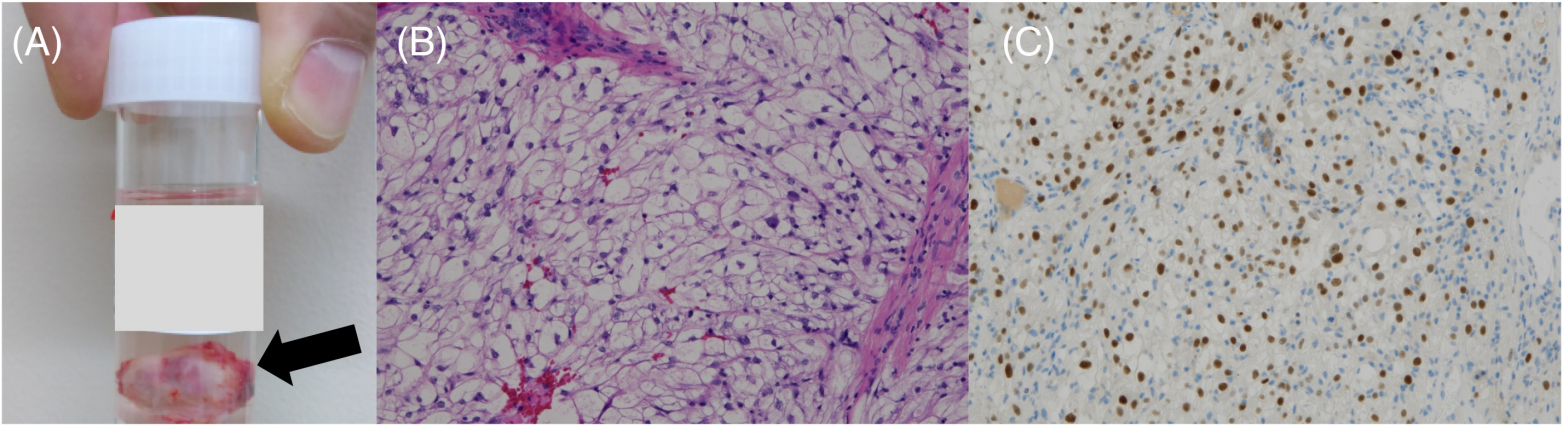
(A) Specimen from the cryobiopsy (black arrow). The size of the specimen was 17 × 15 × 14 mm. (B) Haematoxylin and eosin and (C) paired box gene (PAX) staining. Samples from the left pleural biopsy, forceps biopsy and cryobiopsy all showed a full structure of atypical cells with clear spores. Immunostaining was positive for PAX8, CD10, and carbonic anhydrase IX and negative for thyroid transcription factor 1 and oestrogen receptor, consistent with renal cancer metastasis

Pathological examination of the parietal and visceral pleural biopsies yielded a diagnosis of renal cancer metastasis (Figure 3).

## DISCUSSION

We safely performed cryobiopsy under local anaesthesia with thoracoscopy to an intrathoracic mass with negative pleural fluid cytology. Because we suspected primary lung cancer in addition to recurrence of renal and breast cancer, it was necessary to collect a sufficient amount of tissue for possible genetic analysis. Rapid cytology of the forceps biopsy from the wall pleura was negative, so we added forceps biopsy from the visceral pleura and cryobiopsy to obtain sufficient specimens. As a result, we were able to make a diagnosis of renal cancer metastasis.

Analysis of 55 autopsy cases revealed that pleural metastases of malignant tumours were more common in the visceral pleura, with 51 (92.7%) cases in the visceral pleura and 31 (56.4%) cases in the parietal pleura.^3^ Therefore, biopsy from the visceral pleura may improve the diagnostic rate.

There is one previous report of a biopsy from the visceral pleura. Terashita et al. safely performed a forceps biopsy from the visceral pleura in an 82‐year‐old woman with negative pleural fluid cytology and diagnosed lung adenocarcinoma.^2^

We added cryobiopsy because, compared to forceps biopsy, it provides sufficient specimen volume and can reliably collect tumour tissue in masses with a hard surface.

Cryobiopsy from the visceral pleura requires attention to complications such as pneumothorax and haemorrhage due to the collection of large specimens. According to the BTS guidelines, visceral pleural biopsy should be performed by an experienced physician.^1^

To reduce pneumothorax, a previous study reported pressing thoracoscopes and biopsy forceps against the nodule vertically to avoid grasping the normal lung.^2^ In the present study, we selected a convex‐shaped mass in the thoracic cavity; a biopsy specimen was obtained by keeping the side of the cryoprobe tip against the most convex part of the tumour in the thoracic cavity to avoid contact with the normal lung.

In case of bleeding, in addition to thoracoscopic compression, preparation for argon plasma coagulation and the presence of a cardiothoracic surgeon should be considered. The freezing time for cryobiopsy in parietal pleural biopsies in previous studies ranges from 3 to 10 s.^4^ In the present case, the freezing time was 3 s. A shorter freezing time was considered because the biopsy was taken from the visceral pleura and was expected to be haemorrhagic due to metastatic renal cell carcinoma.

We also suggest avoiding the procedure if the mobility of the mass makes it difficult to fix the cryoprobe.

To the best of our knowledge, this is the first report of cryobiopsy of the visceral pleura with thoracoscopy under local anaesthesia. We suggest that this is a safe and useful feasible technique in cases meeting the following criteria: negative pleural fluid cytology, difficulty with general anaesthesia, lack of sufficient prietal pleural winfow for biopsies due to extensive adhesions, negative rapid cytology, and the presence of nodules or masses on the visceral pleura.

## AUTHOR CONTRIBUTIONS

Chie Morita wrote and edited the manuscript. Atsushi Kitamura reviewed the manuscript. All authors performed the thoracoscopy and/or participated in patient care.

## CONFLICT OF INTEREST

None declared.

## ETHICS STATEMENT

The authors declared that appropriate written informed consent was obtained from the patient for the publication of this manuscript and any accompanying images.

## Data Availability

The data that support the findings of this study are available from the corresponding author upon reasonable request.
